# *Citrobacter tructae* sp. nov. Isolated from Kidney of Diseased Rainbow Trout (*Oncorhynchus mykiss*)

**DOI:** 10.3390/microorganisms9020275

**Published:** 2021-01-28

**Authors:** Won Joon Jung, Hyoun Joong Kim, Sib Sankar Giri, Sang Guen Kim, Sang Wha Kim, Jeong Woo Kang, Jun Kwon, Sung Bin Lee, Woo Taek Oh, Jin Woo Jun, Se Chang Park

**Affiliations:** 1 Laboratory of Aquatic Biomedicine, College of Veterinary Medicine and Research Institute for Veterinary Science, Seoul National University, Seoul 08826, Korea; cwj0125@snu.ac.kr (W.J.J.); hjoong@snu.ac.kr (H.J.K.); ssgiri@snu.ac.kr (S.S.G.); imagine0518@snu.ac.kr (S.G.K.); blackcat9201@snu.ac.kr (S.W.K.); kck90@snu.ac.kr (J.W.K.); kjun1002@snu.ac.kr (J.K.); lsbin1129@snu.ac.kr (S.B.L.); mike0202@snu.ac.kr (W.T.O.); 2 Department of Aquaculture, Korea National College of Agriculture and Fisheries, Jeonju 54874, Korea

**Keywords:** *Citrobacter tructae*, rainbow trout, fish pathogen, phylogeny, genome sequence, antibiotic resistance

## Abstract

A novel *Citrobacter* species was isolated from the kidney of diseased rainbow trout (*Oncorhynchus mykiss*) reared on a trout farm. Biochemical characterization and phylogenetic analysis were performed for bacterial identification. Sequencing of the 16S rRNA gene and five housekeeping genes indicated that the strain belongs to the *Citrobacter* genus. However, multilocus sequence analysis, a comparison of average nucleotide identity, and genome-to-genome distance values revealed that strain SNU WT2 is distinct and forms a separate clade from other *Citrobacter* species. Additionally, the phenotype characteristics of the strain differed from those of other *Citrobacter* species. Quinone analysis indicated that the predominant isoprenoid quinone is Q-10. Furthermore, strain virulence was determined by a rainbow trout challenge trial, and the strain showed resistance to diverse antibiotics including β-lactams, quinolone, and aminoglycosides. The complete genome of strain SNU WT2 is 4,840,504 bp with a DNA G + C content of 51.94% and 106,068-bp plasmid. Genome analysis revealed that the strain carries virulence factors on its chromosome and antibiotic resistance genes on its plasmid. This strain represents a novel species in the genus *Citrobacter* for which the name *C. tructae* has been proposed, with SNU WT2 (=KCTC 72517 = JCM 33612) as the type strain.

## 1. Introduction

*Citrobacter* species are Gram-negative coliform bacteria in the phylum Proteobacteria, family Enterobacteriaceae [1]. The cells are long, rod-shaped, and typically 1–5 μm in length, using flagella for motility [2,3]. The genus *Citrobacter* contains 14 species (*C. freundii*, *C. koseri*, *C. amalonaticus*, *C. farmeri*, *C. youngae*, *C. braakii*, *C. werkmanii*, *C. sedlakii*, *C. rodentium*, *C. portucalensis*, *C. europaeus*, *C. pasteurii*, *C. gillenii*, and *C. murliniae*) [4]. Diverse *Citrobacter* species are found in various environments, such as water and soil, and are also present in the animal gut microbiota such as in the human intestine [5,6]. Phylogenetic analysis of several housekeeping genes is typically performed for accurate isolation and identification of bacteria in the genus *Citrobacter* [4]. 

Opportunistic pathogens among these bacteria are very rare; however, a few species, such as *C. freundii*, cause bacteremia in immunosuppressed patients [7]. Furthermore, several clinical cases of neonatal meningitis in humans caused by *C. freundii* were reported [8]. *Citrobacter freundii*, as a pathogenic bacterium in animals, has mostly been studied in trout and cyprinids [9]. *Citrobacter* species exhibit multiple resistance against various antibiotics because of their plasmid-encoded resistance genes [10]. As these bacteria can act as opportunistic pathogens inducing nosocomial infections in both human and animals, their antibiotic resistance mechanisms have been studied, including broad evaluation of *qnrB* and *aac* in the context of infection treatment [7,11,12].

In the current study, we describe a novel strain SNU WT2 which was isolated from a moribund rainbow trout (*Oncorhynchus mykiss*) during an epidemiologic study of the aquatic environment of rainbow trout fisheries in Korea. The isolated bacterium is classified as a novel species in the genus *Citrobacter* based on its phylogenetic, chemotaxonomic, and biochemical analysis results. In addition, the pathogenicity of the strain was measured in a challenge trial on rainbow trout. Genomic characterization using the complete sequence of the strain was performed to detect the presence of virulence factors and antibiotic resistance genes.

## 2. Materials and Methods 

### 2.1. Isolation and Characterization of Bacterial Strain SNU WT2 

Strain SNU WT2 was isolated from a single rainbow trout farm in Chungbuk Province, Korea, in 2018. The farm rears 60,000 rainbow trout in ten water tanks per annum. Each water tank is supplemented with 5–6 tons of ground water, with an average water temperature of 15 °C. The farm provided five fish samples (20 ± 2 g) for the diagnosis of the cause of disease and relatively high mortality observed (mortality of 12–15%, which was higher than the average mortality of the farm (<5%). 

The fish first underwent postmortem microscopic examination for the presence of fungal and parasitic infections. The gills and fins were swabbed and smeared on a glass slide for inspection under a light microscope. Because of the relatively low mortality, the fish were screened for the presence of only the main viral diseases of salmonids by PCR (infectious hematopoietic necrosis virus, infectious pancreatic necrosis virus, and viral hemorrhagic septicemia virus) [13]. 

For bacterial analysis, the liver, spleen, and kidney of moribund fish were separately collected and homogenized in 300 μL of sterile phosphate-buffered saline (PBS). After homogenization, 100 μL of the suspension was used for bacterial cultivation. Strain SNU WT2 was isolated from the kidney homogenate after 48 h cultivation on a tryptic soy agar (TSA; BD Difco, Detroit, MI, USA) at 25 °C. For long-term storage, the strain was preserved in 25% (vol/vol) glycerol at −80 °C. The cells from pure colonies grown in TSA for 24 h were used for morphological and biochemical characterization. Morphological analysis was performed by using transmission electron microscopy (80 kV) (JEM1010; JEOL, Akishima, Japan). Gram staining was performed by using a Gram-staining kit (bioMérieux®, Marcy-l’Étoile, France). Oxidase activity was tested with 1% tetramethyl *p*-phenylenediamine (Merck, Kenilworth, NJ, USA), and catalase activity was evaluated in the presence of 3% (*v*/*v*) aqueous hydrogen peroxide solution. Growth of the strain was analyzed at temperatures of 0–50 °C in tryptic soy broth (TSB; BD Difco) after 48 h of incubation. Furthermore, NaCl tolerance was evaluated in TSB supplemented with 0%, 2%, 4%, 6%, 8%, or 10% at 25 °C for 48 h in a shaking incubator. To determine the pH range for growth, the pH of the TSB medium was adjusted with 0.1 M HCl and 0.1 M NaOH to a value of 4.0–11.0 at 1.0 pH unit intervals. To verify the strain growth under anaerobic conditions, the cells were cultured in TSB-containing tubes blocked with paraffin at 25 °C for 48 h. For biochemical characterization, the phenotypic characteristics of the strain were evaluated by using API 50 CH (bioMérieux) and API 20E strips (bioMérieux) incubated at 25 °C for 24 h. For comparative analysis, the biochemical characteristics of strain SNU WT2 were compared with those of closely related *Citrobacter* species: *C. freundii* ATCC 8090, *C. braakii* ATCC 51113, and *C. werkmanii* CIP 104555.

### 2.2. Phylogenetic Analysis and Genome Sequencing of Strain SNU WT2 

For bacterial identification, the total genomic DNA was extracted from pure cultured colonies. The cells were suspended in 300 μL of Tris-EDTA (TE) buffer, heated at 100 °C for 20 min, and centrifuged at 8000× *g* for 10 min. The pellet was discarded, and 100 μL of the supernatant was used for polymerase chain reaction (PCR). For 16S rRNA gene sequencing, universal primers (27F and 1492R) targeting the gene were used [14]. To identify the species of the strain, five fragments of protein-encoding housekeeping genes (*gyrB* (DNA gyrase), *recN* (DNA repair), *rplB* (ribosomal protein L2), *fusA* (elongation factor G), and *leuS* (tRNA synthetase)) were used. The fragments were PCR-amplified as described previously [2,4,15]. For gene sequence analysis, the PCR products were submitted to the genomic division of Macrogen (Seoul, Korea), where nucleotide sequencing reaction was performed using an ABI PRISM 3730XL analyzer and BigDye^®^ Terminator v. 3.1 Cycle Sequencing kit (Applied Biosystems, Foster City, CA, USA). In previous studies, identification of all *Citrobacter* sp. at the species level was not possible using biochemical methods, MALDI-TOF MS, or sequencing of the 16S rRNA gene. However, analysis of the *recN* sequences precisely distinguished the *Citrobacter* species. Therefore, we performed phylogenetic analysis based on *recN* alone [2,4,16]. The remaining four gene fragments were concatenated and used in a multilocus sequence analysis [4,17]. The aligned sequences were edited using BioEdit software. The sequence of the 16S rRNA gene was compared with other available 16S rRNA gene sequences by National Center for Biotechnology Information (NCBI) BLASTn searching and with data in the EzBioCloud server (https://www.ezbiocloud.net/ accessed on 10 October 2020) to identify related strains. Phylogenetic trees were constructed using the neighbor-joining method, and genetic distances were estimated using Kimura’s 2-parameter model [18,19]. The tree topology was evaluated using bootstrap analysis with 1000 replicates [20]. 

**Figure 1:**
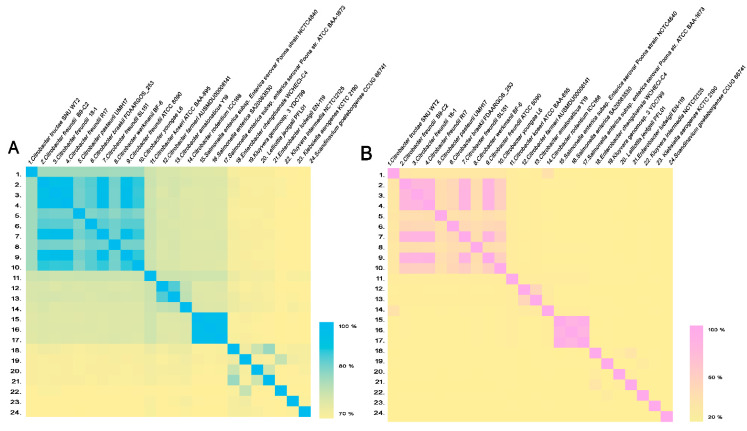
Heat map of average nucleotide identity (ANI) values and genome-to-genome distance calculator (GGDC) values compared among 24 related strains. (**A**) ANI heat map. (**B**) GGDC heat map. ANI and GGDC values are indicated by the color intensity. The strain numbers in Figure 1 are the same as that in Table 1.

The genome of strain SNU WT2 was sequenced using a hybrid approach involving a PacBio RS II system (Pacific Biosciences, Menlo Park, CA, USA) and HiSeq 2000 platform (Illumina). After complete genome sequencing and assembly, the average nucleotide identity (ANI) and genome-to-genome distance were calculated. ANI was calculated using the OrthoANIu tool (https://www.ezbiocloud.net/tools/ani accessed on 10 October 2020) [21], and genome-to-genome distance calculation (GGDC) was conducted using the tool formula 2 available at DSMZ (http://ggdc.dsmz.de/distcalc2.php accessed on 10 October 2020) (Table 1) [22,23,24]. To determine the correlation pattern between species and strains based on their ANI and GGDC values of Table 1, heat maps were drawn (Figure 1).

### 2.3. Chemotaxonomic Analysis 

For fatty acid methyl ester analysis, the strain was cultured on TSA plates at 25 °C for 48 h. Fatty acids and esters were extracted according to the instructions of the Sherlock Microbial Identification System and were analyzed using a Hewlett Packard HP 6890 and Microbial Identification software [25,26]. The analysis was performed at the Korean Culture Center of Microorganisms (KCCM). 

The strain was cultured in TSB at 25 °C for 24 h, and quinone analysis (HPLC), polar lipid identification, and diaminopimelic acid (DAP) analysis were performed at KCCM. For quinone analysis, the cultured strain was freeze-dried. Quinone was extracted with chloroform-methanol (2:1, *v*/*v*), after which the sample was filtered through Whatman No. 2 filter paper. This sample was concentrated using a vacuum centrifuge and then mixed with 100 μL of chloroform-methanol (8.5: 1.5, *v*/*v*) and centrifuged at 18,472× *g* for 5 min. The supernatant was used for HPLC analysis. 

For polar lipid analysis, the strain was harvested from TSB, washed 2–3 times with distilled water, and freeze-dried. Next, 50 mg of the freeze-dried sample was added to a screw-capped tube and mixed well for 15 min after adding 2 mL of methanol–0.3% NaCl solution (100:10) and 2 mL of hexane. Centrifugation at 15,928× *g* for 10 min was performed to remove the supernatant, and then, 1 mL of hexane was added and mixed well, followed by centrifugation at 15,928× *g* to remove the top layer. The remaining bottom layer was sealed with parafilm, heated at 100 °C for 5 min, and then cooled at 37 °C for 5 min. Next, 2.3 mL of chloroform–methanol–0.3% NaCl solution (*w*/*v*) at a ratio of 90:100:30 (*v*/*v*) was added, stirred for 1 h, mixed well, and centrifuged at 15,928× *g*, and the top layer was transferred to another tube. The chloroform–methanol–0.3% NaCl solution (*w*/*v*) at a ratio of 50:100:40 (*v*/*v*) (0.75 mL) was added to the remaining bottom layer, stirred for 30 min, mixed well, and centrifugated; the top layer was added to the previously separated upper layer. Chloroform and the 0.3% NaCl solution were added to the solution and centrifuged at 15,928× *g*. After removing the upper layer, the lower layer was centrifuged to dry the upper layer in a rotary evaporator. Finally, the sample was dissolved in 0.3 mL of distilled water for thin-layer chromatography (TLC) analysis. A standard was prepared to compare the location of the spot; we placed 10 μL of the sample in a 1.5 cm offset corner on the bottom-left side of the High Performance Thin-Layer Chromatography (HPTLC) plate (10 × 10 cm, Merck 5631) and dried the plate. The dried TLC plate was developed in the primary direction in chloroform–methanol–water solvent at a ratio of 65:25:3.8 (*v*/*v*) and dried for at least 30 min. This TLC plate was developed in the secondary direction under a chloroform–methanol–acetic acid–water solvent at the ratio 40:7.5:6:1.8 (*v*/*v*). The standard and sample plates were prepared under the same conditions. The plates were dried in a hood, sprayed evenly with 5% ethanolic molybdatophosphoric acid, and placed in an oven at 100 °C for approximately 4 min. The position of the spot on the plate compared to that on the standard was determined (the total lipid appears as a black spot on a light green background).

DAP analysis of the strain was performed as follows. The sample was freeze-dried after incubation for 48–72 h in TSB medium; 20 mg of the freeze-dried specimen was added to a screw-capped tube, and then, 1 mL of 6N HCl was added for hydrolysis at 100 °C for 18 h. After cooling at 25 °C, the impurities were filtered out using a filter paper. The solution was transferred to a new tube to dry with nitrogen gas; 0.5 mL of distilled water was added, and the sample was washed and then dried with nitrogen gas. After repeating the above procedure three times, the sample was dissolved in 0.3 mL of distilled water and analyzed. α, ε-diaminopimetic acid (Sigma, St. Louis, MO, USA, 1377) at 1 mg/mL concentration was used as a standard. Dried samples and the standard solution (5 µL each) were placed at approximately 2.5 cm above the baseline of the cellulose TLC plate (20 × 20 cm, Merck 5565). The samples on the TLC plate were separated in a MeOH–H_2_O–10N HCl–pyridine solvent at 80:26:4:10, *v*/*v*. The plate was dried in a hood, sprayed with 0.2% ninhydrin solution in acetone, and placed in a 100 °C oven for approximately 5 min. The position of the dark yellow spot on the plate was compared to that on the standard to detect the DAP isomer.

### 2.4. Antibiotic Susceptibility Testing 

Pure colonies of strain SNU WT2 were used for antibiotic resistance testing. A standard disk diffusion test was performed on a Muller Hinton Agar (BD Difco). Except for the temperature, whole experiment conditions were performed following the Clinical and Laboratory Standards Institute (CLSI) guidelines. We conducted antimicrobial susceptibility testing not at 35 ± 2 °C (as suggested by the CLSI) but at 25 °C, as this is optimal growth temperature of *C. tructae*. The results were interpreted according to the CLSI guidelines. *Escherichia coli* ATCC 25922 was used as the quality control strain [27]. The following antibiotics were tested: ampicillin, piperacillin, ampicillin-sulbactam, piperacillin-tazobactam, cefazolin, cefepime, cefotaxime, cefoxitin, cefuroxime, ceftazidime, ceftizoxime, cefixime, aztreonam, imipenem, meropenem, gentamicin, amikacin, kanamycin, streptomycin, tetracycline, doxycycline, ciprofloxacin, levofloxacin, nalidixic acid, trimethoprim-sulfamethoxazole, trimethoprim, and chloramphenicol.

### 2.5. Bacterium Challenge Trial 

As the clinical strain SNU WT2 was isolated from a cultured moribund rainbow trout, a bacterium challenge was performed to verify the pathogenicity of the strain. Rainbow trout (average weight of 20 g, 13 cm) were purchased from a rainbow trout farm located in Gangwon Province (Korea), which was a different farm from which the strain SNU WT2 was isolated. The fish were maintained at 15 °C in water for 2 weeks before the challenge. Bacteria were grown in TSB at 25 °C for 24 h and washed in PBS before injection into the fish. The bacterial density was determined based on the optical density by using a SmartSpec™ 3000 spectrophotometer (Bio-Rad, Hercules, CA, USA). The cells were diluted with PBS to 4 × 10^7^, 4 × 10^6^, 4 × 10^5^, and 4 × 10^4^ colony-forming units (CFU) per 100 μL, and 100 μL suspensions were intraperitoneally injected into the fish. The experiments were simultaneously performed in triplicate; each treatment group consisted of 10 fish maintained in a 120 L water tank. The control groups were injected with 100 μL of PBS and treated the same as the experimental groups. Water temperature was maintained at 15 °C, which was the same as the water temperature at the farm from which the bacterial strain was isolated. Every fish group was individually aerated and observed for clinical signs or abnormal behaviors. The experiment was performed for 15 days to determine fish mortality. The bacteria were re-isolated from the fish that died during the experiment to fulfill the Koch’s postulate regarding bacterial pathogenicity.

### 2.6. Histopathological Analysis 

The same fish described in Section 2.5 were used for histopathological analysis. As postmortem changes in fish can impact the analysis results, the dead fish were exempt from analysis. Tissue samples (including the kidney, liver, and spleen) were collected and fixed in 10% neutral-buffered formalin. The fixed tissues were sliced and dehydrated in ethanol. The samples were embedded in paraffin, sectioned, stained with hematoxylin and eosin, and observed under a light microscope. Each slide was digitally scanned by Xenos, Inc. (Suwon, Korea). 

### 2.7. Genome Analysis of Strain SNU WT2

The complete genome sequence of strain SNU WT2 was used to detect genes potentially related to virulence factors and antibiotic microbial resistance. The presence of antibiotic resistance genes was investigated using the ARG ANNOT database (http://en.mediterranee-infection.com/articlc.php?laref=283&titre=arg-annot- accessed on 21 September 2020 and BLASTn option in BioEdit software for comparisons and similarity-value calculations. The same strategy was used to identify potential virulence genes, with virulence factor database searching (http://www.mgc.ac.cn/VFs/ accessed on 18 November 2020). The maximum expected value was fixed at 0.0001 for both analyses.

## 3. Results and Discussion

### 3.1. Phylogenetic and Genome Analysis of Strain SNU WT2

**Figure 2:**
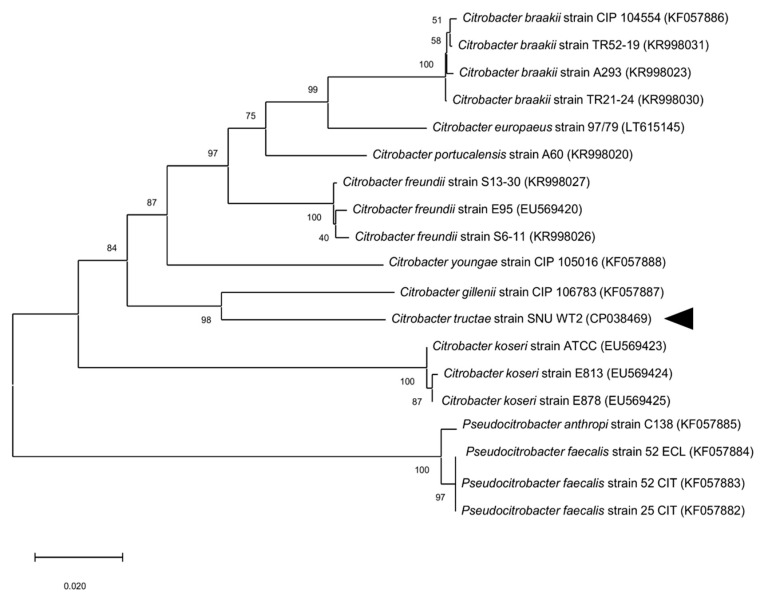
Phylogenetic tree based on the *recN* gene of *Citrobacter* species: the neighbor-joining method was used for the tree construction in MEGA 7.0 [28]. The bootstrap values obtained after 1000 replicates are provided at the nodes. Bar, 0.02 changes per nucleotide position. The arrow head indicates SNU WT2.

**Figure 3:**
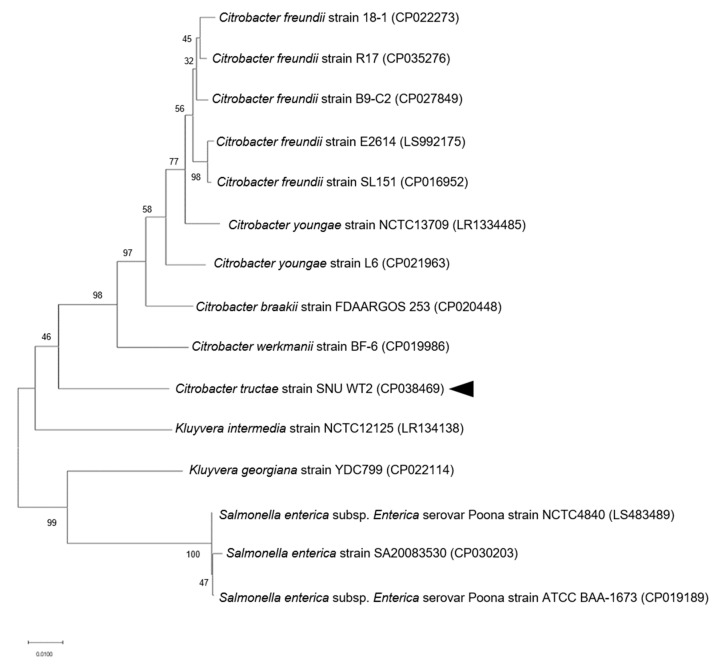
Phylogenetic tree based on four housekeeping genes (*gyrB* (DNA gyrase), *rplB* (ribosomal protein L2), *fusA* (elongation factor G), and *leuS* (tRNA synthetase)) of the *Citrobacter* species: the maximum-likelihood method was used for the tree construction in MEGA 7.0 [29]. The bootstrap values obtained after 1000 replicates are provided at the nodes. Bar, 0.01 changes per nucleotide position. The arrow head indicates SNU WT2.

The 16S rRNA gene of strain SNU WT2 (GenBank accession number: MN093886) was most closely related to *C. portucalensis* [2]. However, the *recN* sequence, which is typically used for distinguishing species in the genus *Citrobacter*, revealed that the strain was highly similar to *C. gillenii* (89.63% similarity). The similarity value was relatively low, and the phylogenetic tree constructed based on *recN* revealed that strain SNU WT2 (GenBank accession number: MN107009) was related to *C. gillenii* rather than to *C. portucalensis* (Figure 2). Because of these contradictory results, another phylogenetic tree was constructed using four housekeeping genes for multilocus sequence analysis (*fusA*, *leuS*, *rplB*, and *gyrB* (GenBank accession numbers: MN107004, MN107005, MN107006, and MN107008)). The analysis indicated that strain SNU WT2 formed a new single clade, different from that of the original *Citrobacter* genus, suggesting that the strain was a novel subspecies in the *Citrobacter* group (Figure 3). To confirm the species assignment of strain SNU WT2, complete whole-genome sequencing was performed and the obtained sequence was compared with that of other *Citrobacter* species. The draft SNU WT2 genome was a circular chromosome of 4,840,504 bp, with a 51.94% G + C content. The calculated ANI values were below the 94–96% cutoff value proposed for species delimitation; the highest value for strain SNU WT2 was that with *C. freundii* strain B9-C2 (87.43%). In addition, the GGDC values were below the 70% species boundary recommended previously, and the highest value obtained was between that of strain SNU WT2 and *C. freundii* strain B9-C2 (33.4%) (Table 1). These observations indicate that strain SNU WT2 represents a putative novel species in the genus *Citrobacter*.

### 3.2. Description of Strain SNU WT2 Citrobacter tructae sp. nov.

Round, convex, and whitish colonies with a diameter of 0.5–1.0 mm were predominant on TSA after 48 h of incubation at 25 °C. As determined by morphological analysis using transmission electron microscopy, the cells were 1–2 μm wide and 1–2 μm long. The cells were aerobic, Gram-negative, and motile. The strain tested positive for oxidase and catalase activity and was able to grow at 4–45 °C, with an optimal growth temperature of 25 °C. Furthermore, the strain grew in a pH range of 5–9 and tolerated 0–6% NaCl. Phenotypic characteristic testing using API 50 CH strips revealed that the SNU WT2 activity differed from that of *C. freundii* ATCC 8090 in reactions with esculin, cellobiose, saccharose, β-gentiobiose, D-lyxose, 2-keto-gluconate, ornithine decarboxylase, and indole production. Furthermore, according to API 20E strip analysis, the strain showed different activities from those of *C. braakii* ATCC 51113 with respect to H_2_S production, indole production, amygdalin fermentation, and the presence of cytochrome oxidase. Furthermore, strain SNU WT2 showed different activities compared to those of its closest relative (see Section 3.2.), *C. gillenii* DSM 13694, concerning H_2_S production, acid production from esculin and melibiose, and β-galactosidase activity. The major fatty acids of strain SNU WT2 were C16:0 (30.63%), cyclo-C17:0 (26.27%), cyclo-C19:0 ω8c (9.86%), and C14:0 (9.34%). Polar lipid analysis revealed the presence of phosphatidylethanolamine, phosphatidylglycerol, and diphosphatidylglycerol as the major components (Figure S1). The predominant isoprenoid quinone of strain SNU WT2 was Q-10, and DAP analysis confirmed the presence of *meso*-DAP in the cell wall.

### 3.3. Multiple Antibiotic Resistance of Strain SNU WT2

Antibiotic resistance analysis suggested that strain SNU WT2 was resistant to diverse antibiotics, similar to other *Citrobacter* species [7,30]. It was not susceptible to any of the antibiotics examined; it tested intermediate for cefoxitin, imipenem, meropenem, ciprofloxacin, and levofloxacin. Similar to other *Citrobacter* species, strain SNU WT2 carries a plasmid (106,068 bp) which may contribute to the observed resistance. The strain was isolated from a moribund rainbow trout, indicating that it causes disease in fish. The observed multidrug resistance may be associated with considerable economic losses in rainbow trout fisheries.

### 3.4. Pathogenicity of Strain SNU WT2 and Histopathological Findings

To examine the pathogenicity of strain SNU WT2, the 50% lethal dose (LD_50_) was determined in a challenge trial. No mortality was observed in any fish groups at 5 days after artificial infection. Fish death was observed on day 6 in groups infected with 4 × 10^7^, 4 × 10^6^, and 4 × 10^5^ CFU/fish and on day 7 in a group infected with 4 × 10^4^ CFU/fish. The most rapid mortality rate was apparent among fish infected with 4 × 10^7^ CFU/fish. The calculated LD_50_ value was 7.3 × 10^6^ CFU/fish, which was lower than that of *C. freundii* [31]. As the LD_50_ value exceeded 10^6^ CFU/fish, the bacterium cannot be considered a serious fish pathogen. Nevertheless, it may be an opportunistic pathogen of rainbow trout. 

**Figure 4:**
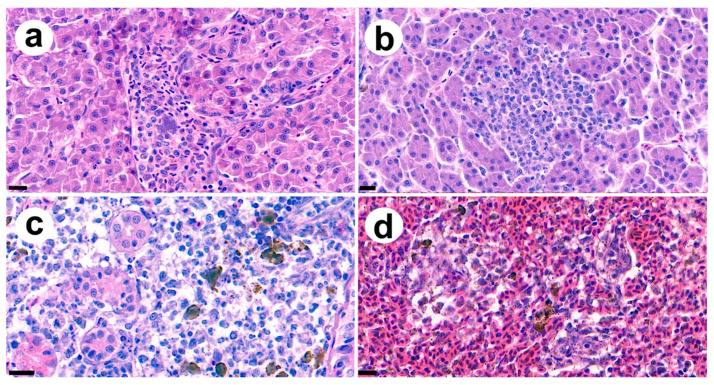
(**a**) Bacteremia observed on the hepatic vein of infected fish (bar indicating 20 μm) and vacuolation can be observed on surrounding hepatocyte. (**b**) Moderate focal to coalescing hepatic necrosis was observed on the liver of infected fish (bar indicating 20 μm). (**c**) Hyaline droplets accumulated on renal tubule epithelial cells with macrophages and melanocytes observed around the damaged tubule (bar indicating 20 μm). (**d**) Coagulative necrosis was observed in spleen with a large amount of cellular debris accompanied by free bacterial rods and macrophages surrounding the area (bar indicating 10 μm).

Histopathological analysis revealed major lesions induced by the infection. Among the main commonly observed lesions was bacteremia of the liver (Figure 4a), which occurred surrounding hepatocyte necrosis in a multifocal area (Figure 4b). In addition, in the kidney, multiple hyaline droplet accumulations in the tubular epithelium were observed, with infiltration of mononuclear cells and macrophages surrounding the infected tubules (Figure 4c). Further, signs of peritonitis in the spleen, including macrophage infiltration, were apparent with a necrotizing area showing lesions on the liver (Figure 4d). Overall, the infection affected and damaged the major organs (liver, spleen, and kidney) with bacteremia, which was also observed in the hepatic vein of diseased fish.

### 3.5. Genome Features of Strain SNU WT2

The complete genome of strain SNU WT2 is a single circular chromosome of 4,840,504 bp (GenBank accession number: CP038469) and plasmid of 106,068 bp (GenBank accession number: CP038468). The chromosome encodes 4430 coding regions, and 83 tRNA and 25 rRNA genes, with 51.9 GC%; the plasmid encodes 119 coding regions, with 52.3 GC%. Genes related to antibiotic resistance are mostly located on the plasmid, and only two genes were detected on the chromosome (genes encoding β-lactamase and related to penicillin-binding protein) (Table 2). The plasmid harbors genes involved in resistance to tetracycline, streptomycin, β-lactams, chloramphenicol, kanamycin, and neomycin. Considering the antibiotic susceptibility data (Section 3.3), strain SNU WT2 most likely became multi-antibiotic-resistant after acquiring the plasmid. In addition, genes related to virulence factors previously identified in other *Citrobacter* species were detected using same blast searching method compared to database listed in http://www.mgc.ac.cn/VFs/ accessed on 15 December 2020. The detected virulence genes of strain SNU WT2 are described in Table 3. The virulence factors of Table 3 might be related to the pathogenicity of strain SNU WT2, as demonstrated by the challenge trial. Specific correlations between these virulence factors and pathogenicity should be verified through further studies. 

## 4. Conclusions

Based on phylogenetic, biochemical, chemotaxonomic, and genome analyses, the strain SNU WT2 is considered a novel species within the genus *Citrobacter*, with the proposed name *C. tructae* (truc’tae L. gen. n. tructae of a trout). The phylogenetic analysis revealed that the strain cannot be distinguished from other *Citrobacter* species based on the *recN* sequence. Furthermore, phylogenetic analysis using the 16S rRNA gene or other housekeeping genes yielded no relevant results related to species identification of this strain, as the multilocus sequence analysis (MLSA) results showed separate clades not belonging to the original *Citrobacter* groups. However, an analysis of the ANI and GGDC values clearly indicated that strain SNU WT2 is a novel species in the *Citrobacter* genus. The strain SNU WT2 (=KCTC 72517 = JCM 33612) was isolated from the kidney of a diseased rainbow trout in Korea. The DNA G + C content of the type strain is 51.94%. The strain pathogenicity in rainbow trout was confirmed in a challenge trial; high strain doses resulted in fish mortality. Histopathological analysis of the bacterial pathogenicity revealed several lesions on the liver and kidney of infected fish, which may have been the cause of death in the challenge trial. An analysis of the complete genome sequence of strain SNU WT2 was performed considering the strain’s pathogenicity and antibiotic resistance. Diverse antibiotic resistance genes and virulence factors were detected on the chromosome and plasmid. The confirmed virulence and resistance to diverse antibiotics may cause appreciable problems in the rainbow trout fisheries in the near future, notably because the strain was susceptible to none of the antibiotics tested in the current study. Further research is required to determine the appropriate treatment of infections caused by this bacterium.

## Figures and Tables

**Table 1:** Average nucleotide identity and genome-to-genome distance calculator (GGDC) analysis for *Citrobacter tructae* SNU WT2 and other related species.

Number	Species	Strain	*C. tructae* SNU WT2 (ANI Value %)	*C. tructae* SNU WT2 (GGDC Value %)
1.	*Citrobacter tructae*	SNU WT2	100	100
2.	*C* *itrobacter freundii*	B9-C2	87.43	33.40
3.	*C* *itrobacter freundii*	18-1	87.38	33.20
4.	*C* *itrobacter freundii*	R17	87.35	33.1
5.	*C* *itrobacter pasteurii*	UMH17	87.35	33.2
6.	*C* *itrobacter* *braakii*	FDAARGOS_253	87.34	33.1
7.	*C* *itrobacter freundii*	SL151	87.33	33.2
8.	*C* *itrobacter werkmanii*	BF-6	87.25	32.7
9.	*C* *itrobacter freundii*	ATCC 8090	87.23	33.1
10.	*C* *itrobacter youngae*	L6	87.14	33.1
11.	*C* *itrobacter koseri*	ATCC BAA-895	83.49	27.1
12.	*C* *itrobacter farmeri*	AUSMDU00008141	82.46	25.3
13.	*C* *itrobacter amalonaticus*	Y19	82.44	25.6
14.	*C* *itrobacter rodentium*	ICC168	82.06	37.6
15.	*Salmonella enterica* subsp. *Enterica* serovar Poona	NCTC4840	81.79	24.8
16.	*Salmonella enterica*	SA20083530	81.73	24.8
17.	*Salmonella enterica* subsp. *Enterica* serovar Poona	ATCC BAA-1673	81.6	24.8
18.	*Enterobacter chengduensis*	WCHECl-C4	79.65	22.9
19.	*Kluyvera* genomosp. *3*	YDC799	79.34	23.1
20.	*Lelliottia jeotgali*	PFL01	79.3	22.9
21.	*Enterobacter ludwigii*	EN-119	79.28	22.6
22.	*Kluyvera intermedia*	NCTC12125	78.77	23
23.	*Klebsiella aerogenes*	KCTC 2190	78.6	22.2
24.	*Scandinavium goeteborgense*	CCUG 66741	78.38	22.3

**Table 2:** Genes of the strain SNU WT2 related to antibiotic resistance compared with information deposited in the ARG-ANNOT database [32].

Query ID	Database ID	Gene Function	% Identity	Alignment Length	Mismatches	QSS ^A^	QSE ^B^	DSS ^C^	DSE ^D^	E-Value	Bit Score
**CP038469**	(Bla)CMY-74:JX440349:1027-2172:1146	AmpC beta-lactamase CMY-74	88.88	1142	127	2,937,846	2,938,987	1	1142	0	1257
**CP038469**	(Bla)CMY-44:FJ437066:1-1134:1134	class C beta-lactamase CMY-44	88.55	926	106	2,937,846	2,938,771	1	926	0	995
**CP038469**	(Bla)CFE-1:AB107899:1008-2153:1161	AmpC beta-lactamase CFE-1	88.35	1142	133	29,378	2,938,987	1	1142	0	1209
**CP038469**	(Bla)CMY-48:HM569226:1040-2185:1146	AmpC beta-lactamase CMY-48	87.96	1146	138	2,937,846	2,938,991	1	1146	0	1178
**CP038469**	(Bla)CMY-13:AY339625:3641-4786:1146	class C beta-lactamase CMY-13	87.52	1146	143	2,937,846	2,938,991	1	1146	0	1138
**CP038469**	(Bla)CMY-5:Y17716:2374-3519:1146	beta-lactamase CMY-5	87.43	1146	144	2,937,846	2,938,991	1	1146	0	1130
**CP038469**	(Bla)LAT-1:X78117:122-1287:1146	beta-lactamase precursor blaLAT-1	87	1146	149	2,937,846	2,938,991	1	1146	0	1090
**CP038469**	(Bla)BIL-1:X74512:127-1272:1146	beta-lactamase bla BIL-1	86.65	1146	153	29,378	29,389	1	1146	0	1059
**CP038469**	(Bla)Penicillin_Binding_Protein_*Ecoli*: CP002291:6 64439-666340:1902	Penicillin-binding protein 2 mrdA	82.35	1898	335	1,975,761	1,977,658	1	1898	0	1106
**CP038469**	(Bla)AmpH:CP003785:4208384-4209544:1161	Penicillin-binding protein AmpH	81.62	729	134	2,267,261	2,267,989	385	1113	3 × 10^−103^	383
**CP038469**	(Bla)AMPH_Ecoli:AP012030:395554-396711:1158	Beta-lactamase class C and penicillin binding proteins	80.49	687	134	2,266,877	2,267,563	1	687	4 × 10^−78^	299
**CP038468**	(Tet)TetD:AB089602:1521-2705:1185	tetracycline resistant tetD	100	1185	0	47,708	48,892	1	1185	0	2349
**CP038468**	(AGly)StrB:FJ474091:264-1100:837	streptomycin resistance protein B	100	837	0	38,821	39,657	1	837	0	1659
**CP038468**	(Sul)SulII:EU360945:1617-2432:816	SulII gene resistant to beta lactam	100	816	0	37,142	37,957	1	816	0	1618
**CP038468**	(Phe)CatB4:EU935739:59054-59602:549	chloramphenicol acetyltransferase cat B4	100	108	0	34,213	34,320	549	442	4 × 10^−54^	214
**CP038468**	(AGly)Aph3-Ia:HQ840942:23569-24384:816	aphA1a confers resistance to kanamycin and neomycin	99.88	816	1	32,597	33,412	816	1	0	1610
**CP038468**	(AGly)StrA:AB366441:22458-23261:804	streptomycin resistance protein A	99.88	804	1	38,018	38,821	1	804	0	1586
**CP038468**	(Phe)FloR:AKLJ01000508:383-1597:1215	floR	99.84	1215	2	40,613	41,827	1215	1	0	2393

QSS ^A^: query sequence start, QSE ^B^: query sequence end, DSS ^C^: database sequence start, and DSE ^D^: database sequence end.

**Table 3:** Genome fragments related to virulence factors of *Citrobacter* species located on the strain SNU WT2 compared against database of VFDB [33].

Query ID	Database ID	Gene Function	% Identity	Alignment Length	QSS ^A^	QSE ^B^	DSS ^C^	DSE ^D^
**CP038469**	VFG049144	(acrB) acriflavine resistance protein B (AcrAB) (*Klebsiella pneumoniae* subsp. *pneumoniae* NTUH-K2044)	85	1572	2,164,436	2,166,007	1528	3099
**CP038469**	VFG048830	(gnd) 6-phosphogluconate dehydrogenase [capsule) [*Klebsiella pneumoniae* subsp. *pneumoniae* NTUH-K2044)	83	1403	440,817	442,219	1	1403
**CP038469**	VFG001443	(ompA) outer membrane protein A (OmpA) (*Escherichia coli* O18:K1:H7 str. RS218)	89	762	1,696,932	1,697,693	280	1041
**CP038469**	VFG048639	(vipB/tssC) type VI secretion system contractile sheath large subunit VipB (T6SS) *(Klebsiella pneumoniae* subsp. *pneumoniae* HS11286)	83	1175	1,677,973	1,679,147	1529	355
**CP038469**	VFG049018	(rcsB) transcriptional regulator RcsB (RcsAB) (*Klebsiella pneumoniae* subsp. *pneumoniae* NTUH-K2044)	87	623	213,022	213,644	623	1
**CP038469**	VFG048693	(clpV/tssH) type VI secretion system ATPase TssH (T6SS) (*Klebsiella pneumoniae* subsp. *pneumoniae* HS11286)	83	887	1,672,108	1,672,994	1454	568
**CP038469**	VFG000917	(chuA) outer membrane heme/hemoglobin receptor ChuA (Chu) (*Escherichia coli* CFT073)	82	963	684,051	685,013	1947	985
**CP038469**	VFG000923	(fepA) ferrienterobactin outer membrane transporter (enterobactin) (*Escherichia coli* CFT073)	84	777	2,033,028	2,033,804	94	870
**CP038469**	VFG048518	(fepA) outer membrane receptor FepA (Ent) (*Klebsiella pneumoniae* subsp. *pneumoniae* NTUH-K2044)	82	915	2,033,030	2,033,944	111	1025
**CP038469**	VFG002329	(fliG) flagellar motor switch protein G (flagella) (*Yersinia enterocolitica* subsp. enterocolitica 8081)	83	748	1,516,371	1,517,118	244	991
**CP038469**	VFG013064	(shuA) outer membrane heme/hemoglobin receptor ShuA (Shu) (*Shigella dysenteriae* Sd197)	81	963	684,051	685,013	1983	1021
**CP038469**	VFG000462	(csgG) curli production assembly/transport protein CsgG (Agf) (*Salmonella enterica* subsp. *enterica* serovar Typhimurium str. LT2)	82	749	1,596,224	1,596,972	1	749
**CP038469**	VFG044165	(entS) enterobactin exporter, iron-regulated (enterobactin) (*Escherichia coli* CFT073)	80	834	2,022,167	2,023,000	842	9
**CP038469**	VFG048409	(entA) 2,3-dihydroxybenzoate-2,3-dehydrogenase (Ent) (*Klebsiella pneumoniae* subsp. *pneumoniae* NTUH-K2044)	84	548	2,016,160	2,016,707	786	239
**CP038469**	VFG048429	(entE) enterobactin synthase subunit E (Ent) (*Klebsiella pneumoniae* subsp. *pneumoniae* NTUH-K2044)	80	902	2,017,805	2,018,706	1589	688
**CP038469**	VFG000925	(fepC) ferrienterobactin ABC transporter ATPase (enterobactin) (*Escherichia coli* CFT073)	82	689	2,025,211	2,025,899	100	788
**CP038469**	VFG002356	(flhA) flagellar biosynthesis protein FlhA (flagella) (*Yersinia enterocolitica* subsp. *enterocolitica* 8081)	80	927	1,461,459	1,462,385	975	49
**CP038469**	VFG048683	(hcp/tssD) type VI secretion system protein, Hcp family (T6SS) (*Klebsiella pneumoniae* subsp. *pneumoniae* HS11286)	84	465	1,673,742	1,674,206	465	1
**CP038469**	VFG000446	(fimD) usher protein FimD (type 1 fimbriae) (*Salmonella enterica* subsp. *enterica* serovar Typhimurium str. LT2)	82	643	2,096,402	2,097,044	1124	482
**CP038469**	VFG000932	(entE) 2,3-dihydroxybenzoate-AMP ligase component of enterobactin synthase multienzyme complex (enterobactin) (*Escherichia coli* CFT073)	84	458	2,017,799	2,018,256	1598	1141
**CP038469**	VFG048797	(ugd) UDP-glucose 6-dehydrogenase (capsule) (*Klebsiella pneumoniae* subsp. *pneumoniae* NTUH-K2044)	80	733	442,849	443,581	428	1160
**CP038469**	VFG002331	(fliI) flagellum-specific ATP synthase FliI (flagella) (*Yersinia enterocolitica* subsp. *enterocolitica* 8081)	80	695	1,518,261	1,518,955	406	1100
**CP038469**	VFG048478	(fepG) iron-enterobactin transporter permease (Ent) (*Klebsiella pneumoniae* subsp. *pneumoniae* NTUH-K2044)	79	862	2,024,255	2,025,115	133	993
**CP038469**	VFG000928	(fepG) iron-enterobactin ABC transporter permease (enterobactin) (*Escherichia coli* CFT073)	82	512	2,024,204	2,024,715	82	593
**CP038469**	VFG048459	(ybdA) enterobactin exporter EntS (Ent) (*Klebsiella pneumoniae* subsp. *pneumoniae* NTUH-K2044)	81	564	2,022,446	2,023,008	563	1
**CP038469**	VFG000930	(entF) enterobactin synthase multienzyme complex component, ATP-dependent (enterobactin) (*Escherichia coli* CFT073)	82	482	2,028,929	2,029,410	2279	1798
**CP038469**	VFG004125	(csgD) DNA-binding transcriptional regulator CsgD (curli fibers/thin aggregative fimbriae (AGF)) (*Salmonella enterica* subsp. *enterica* serovar Typhimurium str. LT2)	86	329	1,594,712	1,595,040	1	329
**CP038469**	VFG000460	(csgE) curli production assembly/transport protein CsgE (Agf) (*Salmonella enterica* subsp. *enterica* serovar Typhimurium str. LT2)	86	321	1,595,439	1,595,759	76	396
**CP038469**	VFG000920	(chuX) putative heme-binding protein ChuX (Chu) (*Escherichia coli* CFT073)	82	458	2,127,828	2,128,285	1	458
**CP038469**	VFG049133	(acrA) acriflavine resistance protein A (AcrAB) (*Klebsiella pneumoniae* subsp. *pneumoniae* NTUH-K2044)	82	449	2,162,149	2,162,597	460	908
**CP038469**	VFG048419	(entB) 2,3-dihydro-2,3-dihydroxybenzoate synthetase, isochroismatase (Ent) (*Klebsiella pneumoniae* subsp. *pneumoniae* NTUH-K2044)	82	473	2,017,165	2,017,637	608	136
**CP038469**	VFG043209	(cheD) methyl-accepting chemotaxis protein CheD (peritrichous flagella) (*Yersinia enterocolitica* subsp. *enterocolitica* 8081)	80	608	2,726,131	2,726,738	1517	910
**CP038469**	VFG048808	(manB) phosphomannomutase (capsule) (*Klebsiella pneumoniae* subsp. *pneumoniae* NTUH-K2044)	85	364	439,986	440,347	715	1076
**CP038469**	VFG002304	(misL) putative autotransporter (MisL) *(Salmonella enterica* subsp. *enterica* serovar Typhimurium str. LT2)	83	413	3,490,020	3,490,431	1880	2291
**CP038469**	VFG048498	(entF) enterobactin synthase subunit F (Ent) (*Klebsiella pneumoniae* subsp. *pneumoniae* NTUH-K2044)	81	471	2,028,580	2,029,050	2625	2155
**CP038469**	VFG000926	(fepD) ferrienterobactin ABC transporter permease (enterobactin) *(Escherichia coli* CFT073)	79	599	2,023,128	2,023,726	22	620
**CP038469**	VFG013067	(shuX) shu locus protein ShuX (Shu) (*Shigella dysenteriae* Sd197)	81	458	2,127,828	2,128,285	1	458
**CP038469**	VFG000933	(entB) isochorismatase (enterobactin) (*Escherichia coli* CFT073)	86	248	2,017,165	2,017,412	608	361
**CP038469**	VFG000918	(chuT) periplasmic heme-binding protein ChuT (Chu) (*Escherichia coli* CFT073)	79	633	2,125,737	2,126,369	271	903
**CP038469**	VFG000924	(fepB) ferrienterobactin ABC transporter periplasmic binding protein (enterobactin) (*Escherichia coli* CFT073)	80	468	2,020,841	2,021,308	73	540
**CP038469**	VFG000919	(chuW) putative oxygen independent coproporphyrinogen III oxidase (Chu) (*Escherichia coli* CFT073)	78	671	2,126,763	2,127,433	286	956
**CP038469**	VFG048468	(fepD) iron-enterobactin transporter membrane protein (Ent) (*Klebsiella pneumoniae* subsp. *pneumoniae* NTUH-K2044)	80	449	2,023,260	2,023,708	142	590
**CP038469**	VFG000461	(csgF) curli production assembly/transport protein CsgF (Agf) (*Salmonella enterica* subsp. *enterica* serovar Typhimurium str. LT2)	81	413	1,595,785	1,596,194	1	413
**CP038469**	VFG000457	(csgB) minor curlin subunit precursor, curli nucleator protein CsgB (Agf) (*Salmonella enterica* subsp. *enterica* serovar Typhimurium str. LT2)	91	155	1,593,794	1,593,948	155	1
**CP038469**	VFG048449	(fepB) iron-enterobactin transporter periplasmic binding protein (Ent) (*Klebsiella pneumoniae* subsp. *pneumoniae* NTUH-K2044)	81	373	2,020,902	2,021,274	134	506
**CP038469**	VFG000931	(entC) isochorismate synthase 1 (enterobactin) (*Escherichia coli* CFT073)	79	598	2,019,406	2,019,944	1188	650
**CP038469**	VFG048990	(galF) UTP-glucose-1-phosphate uridylyltransferase subunit GalF (capsule) (*Klebsiella pneumoniae* subsp. *pneumoniae* NTUH-K2044)	81	380	424,787	425,166	13	392
**CP038469**	VFG002365	(gmd) GDP-mannose 4,6-dehydratase (O-antigen) (*Yersinia enterocolitica* subsp. *enterocolitica* 8081)	78	527	434,829	435,355	268	794
**CP038469**	VFG048885	(gmd) GDP-D-mannose dehydratase (capsule) (*Klebsiella pneumoniae* subsp. *pneumoniae* NTUH-K2044)	78	590	434,577	435,166	25	614
**CP038469**	VFG000936	(iutA) ferric aerobactin receptor precursor IutA (aerobactin) (*Escherichia coli* CFT073)	82	290	546,850	547,139	545	256

QSS ^A^: query sequence start, QSE ^B^: query sequence end, DSS ^C^: database sequence start, and DSE ^D^: database sequence end.

## Data Availability

Data is contained within the article.

## Supplementary Materials

The following are available online at https://www.mdpi.com/2076-2607/9/2/275/s1, Figure S1: Polar lipid analysis of strain SNU WT2 (PE, phosphatidylethanolamine; PG, phosphatidylglycerol; DPG, diphosphatidylglycerol; L, unidentified lipid).
