# Supplementary figures and images for: Citrobacter tructae sp. nov. Isolated from Kidney of Diseased Rainbow Trout (Oncorhynchus mykiss)
Source: Microorganisms. 2021 Jan 28;9(2):275. doi: 10.3390/microorganisms9020275 (PMC7912136; doi:10.3390/microorganisms9020275)

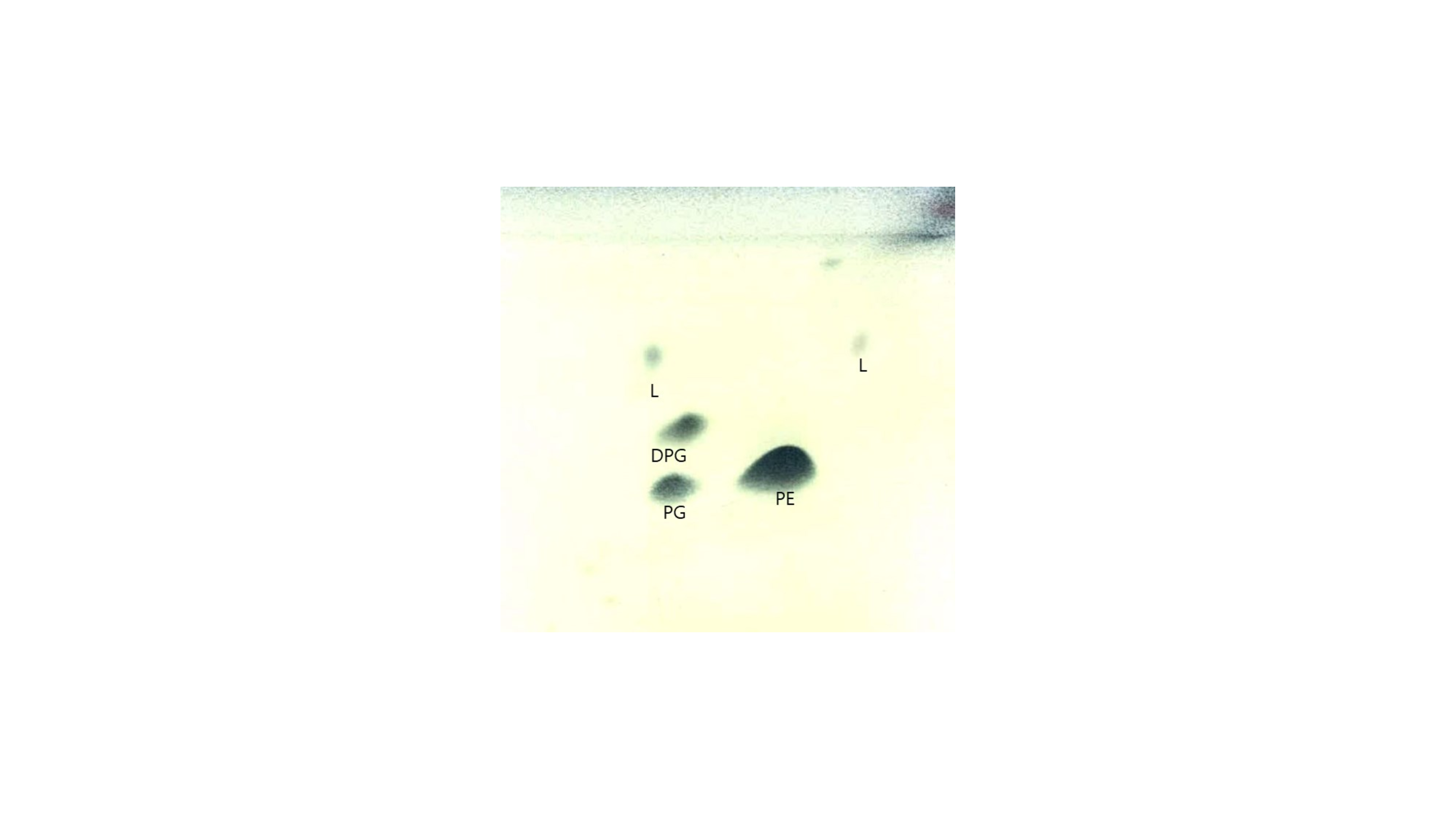

Supplement: Supplementary file 1 [file microorganisms-09-00275-s001.zip › microorganisms-1085415-supplementary.TIF]
